# Using PDX for Preclinical Cancer Drug Discovery: The Evolving Field

**DOI:** 10.3390/jcm7030041

**Published:** 2018-03-02

**Authors:** Juliet A. Williams

**Affiliations:** Novartis Institutes for Biomedical Research, Cambridge, MA 02139, USA; juliet.williams@novartis.com; Tel.: +1-617-871-3763

**Keywords:** patient derived xenograft, avatars, mouse clinical trials (MCT), drug discovery, humanized mouse models

## Abstract

The ability to create patient derived xenografts (PDXs) has evolved considerably from the breakthrough of the development of immune compromised mice. How researchers in drug discovery have utilized PDX of certain cancer types has also changed from traditionally selecting a few models to profile a drug, to opting to assess inter-tumor response heterogeneity by screening across a broad range of tumor models, and subsequently to enable clinical stratification strategies. As with all models and methodologies, imperfections with this approach are apparent, and our understanding of the fidelity of these models continues to expand. To date though, they are still viewed as one of the most faithful modeling systems in oncology. Currently, there are many efforts ongoing to increase the utility and translatability of PDXs, including introducing a human immune component to enable immunotherapy studies.

## 1. Introduction

In the past decade, patient derived tumor xenograft models (PDXs) have increasingly become the preferred tool in research to understand cancer biology, to aid drug discovery, and to translate findings for optimal treatment of the human disease. As with all model systems, PDXs are not perfect, nor are they able to represent the whole plethora of the many cancer types and sub types, but where we stand with our knowledge to date, no other system has been proven to be more, or even equally representative of human cancer. Thus, PDX models remain the chosen way, pre-clinically, to understand many cancers and drug response, in the continued quest to recapitulate human tumors as faithfully as possible. Today, there are thousands of PDX models available with many of them representing particular cancer types; this is important, as the inter-heterogeneity within a specific cancer type and sub-type can be vast and needs to be represented to understand the implications of a biological finding and the impact of a drug response across specific cancer patient populations [1]. 

## 2. Creating and Expanding the Repertoire of PDX

Although the exponential increase using of PDX models in research has been noted in the past decade, the chronicles of cancer research remind us that the transplantation of human cancers into animals has been of interest for over a century [2]. The observation by Murphy in 1913 [3] that serial transplantation of human tumors in chick embryos is possible, while in adult chickens it is not, led to the hypothesis that the adult could possess a defensive mechanism. This subsequently influenced researchers to take the context of the animal host into account and the experimentation of irradiating animals was found to improve take rates and serial transplantation [4], demonstrating the essential role of host immunity in transplantation success. It was the breakthrough of the development of a type of immune-deficient mice, the nude mouse [5], which enabled the creation of xenografts in a high throughput manner. This, timing wise, fit with the increasing of use animals in oncology research, particularly to test drugs for its treatment. Nude mice lack a thymus, and therefore lack functioning T cells [6], but however still possess natural killer (NK) cell activity. Subsequently, more strains of immune-deficient mice have been created, providing addition levels of immunosuppressive ability [7,8,9] and enabling a greater spectrum of patient derived tumor models to be established. Importantly there are now many notable examples of where there are open resources and access to PDX samples: Public Repository of Xenografts PRoXe, EurOPDX, US National Cancer Institute (NCI) repository of patient-derived models, US Pediatric preclinical Testing Consortium (PPTC), Childhood Solid Tumour Network and Children’s Oncology Group (COG) cell culture, and xenograft repository [10,11,12,13,14].

Indeed the representation of cancer as PDX is impressive, despite not being complete [1,15,16]. Notable absents of easily generated models are those representing estrogen-receptor (ER) dependent breast cancer [17] and prostate [18,19]. SCLC PDX models are also a scarce source. There are certain sub-sections of other cancers, which are difficult to grow [20,21] and perhaps not surprisingly it is those of metastatic of origin, and the treatment resistant samples that tend to thrive best in the transplantable animal models [22,23,24] Due to the incomplete representation of human cancers, novel technologies have been emerging to attempt to increase the repertoire to more closely mirror the human disease. To establish ER breast cancer, for example, a technique to inject cells into the ductal cells, rather than the fat pad, has been shown to increase the likelihood of establishing robust ER expressing (and dependent) models [25], although the integrity through passaging still needs to be established. Developing organoids initially for future transplantation, as opposed to directly implanted patient material, has also become a method for enabling PDX to ultimately establish and grow [26].

One technique emerging in the last couple of years has been to use circulating tumor cells (CTCs) as the source. PDXs have also been generated from CTCs that were cultured as organoids [27,28,29]. When directly injected into subcutaneous and bone sites, CTCs have created PDXs, which is often cited as evidence that CTCs contain a subpopulation of metastasis-initiating cells [30,31,32]. Successful PDXs have been established from breast, prostate and small-cell lung cancer CTCs directly implanted into immunocompromised host mice [30,31,33]. Samples with greater numbers of CTCs have higher take rates when xenografted [30,31], which could be due to the increased likelihood that some of the CTCs within a sample will be tumorigenic, as well as the association between high CTC counts and aggressive tumors. With CTCs it might be possible to obtain serial samples to establish PDXs from different stages of disease progression, such as before and after therapeutic resistance as the collection from blood or “liquid biopsies” is non-invasive. Therefore, using CTCs may become an increasingly popular methodology for establishing PDXs, However it is important to note that there are still major challenges to overcome with this technique, such as low concentrations in peripheral blood of patients with different solid tumors, access to technologies to isolate all of the CTCs (both the epithelial and mesenchymal) and general technical challenges [11] In addition, as the numbers of cancer cells shed by tumors can be exceeding low, the biological and clinical relevance has been challenged [34]; how do they capture the tumors heterogeneity and what do they represent is still an ongoing question that needs further investigation.

The host system also plays a role with PDX establishment and the resulting stroma environment [35]. As mentioned, the discovery of nude mice was a prelude to the use of increasingly immunocompromised mouse strains for xenografting [36]. Most PDX have been established in nude mice, but for particular problematic indications, or sub types (such as ER positive breast) more severely immune compromised animals, such as severe combined immune-deficient (SCID) mice are used; these are deficient in both T and B cells [7]. Non-obese diabetic SCID (NOD-SCID) mice are also popular for xenografting some cancers, because they avoid the leaky phenotype of SCID mice (i.e., produce some functioning B and T cells as they age), and also have impaired natural killer (NK) cell function [8,37]. Most recently, NOD-SCID interleukin-2 receptor gamma chain null (NSG) mice have become a common strain used for xenografting; this strain lacks functional T, B, and NK cells [38,39]. Although these hosts are better for the engraftment of some cancer types, this should not be a general assumption for the establishment of all PDXs as many confounding factors are in play [40,41,42]. As the mouse strain used can have a substantial effect on PDX biology [43], each strain of mouse carrying a PDX is a unique model. The choice of the most appropriate model still remains unresolved and should be carefully investigated by the researcher. The costs that are associated with using some of these strains may draw researchers’ preference to the nude mouse if huge benefits in using the more immunecompromised strains are not realized [44]. In addition using immune-compromised hosts with some functioning immune cells enables a model that retains at least some of the stroma-tumor interactions found in a natural setting. Also of note is that mouse lymphomas have been reported to arise from the host after PDX transplantation in the more immune compromised strains, particularly in NOD-SCID mice [45,46,47], sometimes leading to a misdiagnosis of the established model. 

Another systemic feature of host mice that affects the engraftment and growth of hormone-dependent cancers are steroid hormone levels. Supplementing the host mice with estrogen for ER dependent breast PDX take rates has been assumed to maximize the success of PDXs of hormone-dependent cancers, for example, estrogen improves take rates in SCID/Beige mice from 2.4% in the absence of supplementation to 25% in its [44], although may not be necessary to establish all ER dependent tumors *per se*. Similary, testosterone is often used to attempt to increase prostate PDX engraftment [19,44,48,49] In addition, tumors with low take rates as subcutaneous grafts, such as oesophageal, prostate, and low-grade ovarian cancer, often grow more successfully as subrenal capsule [21,43,50] which is assumed to relate to the cells having access a greater blood supply. 

Subcultaneous implanted tumors remain the preferred choice of site, mainly due to the ease of monitoring growth with calipers, but PDXs can also be established orthotopically [21,51,52]. Orthotopic growth kinetics can be monitored using specialist imaging equipment [53], such as ultrasound (US), magnetic resonance imaging (MRI), and positron emission tomography (PET), however for high-throughput studies this specialized monitoring remains inefficient compared to the ease of subcultaneous calipering. Nevertheless, orthotopic grafting may create a host microenvironment that better enables appropriate tissue related gene expression when compared to the subcultaneous site. Orthotopic PDXs may be more likely to metastasize [54,55], and in some cases, more closely reflect patients’ responses to therapy than heterotopic PDXs [56,57]. 

The next generation of PDX models include humanized models to evaluate cancer immunotherapies. An obvious major limitation of PDX tumor models is that because they have to be created in immune compromised mice, many components of the tumor microenvironment are absent. This is important as it has been well documented that the immune system is critically involved in cancer initiation and expansion, which has led to the success and interest in targeting the immune system [58,59]. Consequently, researchers are beginning to explore the use of what is labeled as “humanized-xenograft” models. Humanized-PDX models are created by co-engrafting a patient tumor fragment and human peripheral blood mononuclear cells (PBMC) or hematopoietic stem cells (HSC) into immune-deficient mice [60,61]. 

The development of three different murine strains with IL-2 receptor mutations has increased rates of PBMC and HSC engraftment: NSG, NOG (NOD/SCID/gamma(c)(null)), and BRG (BALB/c-Rag2(null)IL2Rgamma(null)) mice [45,62]. Of these models, NSG lack the IL-2 receptor, whilst NOG and BRG mice express a truncated IL-2 receptor, therefore, all of these models have compromised cytokine signalling and express defective NK cells [62]. To increase the myloid populations, various GEMM mice expressing certain human cytokines have been created. Human versions of genes encoding human MCSF (csf1), human interleukin 3 (IL-3), and GM-CSF, and human thrombopoietin have been generated as a transgenic model in Rag2−/−Il2rg−/− mice (MITRG mice). The resulting human cytokines support the development and function of monocytes, macrophages and NK cells derived from human fetal liver or adult CD34(+) progenitor cells co-injected into the mice [63]. Humanizing PDX has now been demonstrated for many different tumor types [63]. These models are important as they may be able to represent the interplay the various immune components have on each other, and therefore be critical in understanding the translatability of a drug on complex systems. Regardless, many “humanized mice” can be useful depending on the specific question being asked. Although the myeloid fraction is usually under represented, if the T cell population is well modeled, which can be used in drug discovery to identify and rank drug candidates for various immunotherapies, including the clinically successful check point inhibitors, and bispecific molecules carrying the CD3 arm. 

If PBMCs or HSC’s are taken from the same patient the PDX is derived HLA compatibility is ensured. Therefore, these co-engrafted systems could aid the recapitulation of many aspects of the tumor microenvironment [62], and may offer superior models over those in which the allogenic and graft-vs-host phenomena occur. 

## 3. Fidelity and Stability of PDX Models 

As with all model systems, none are perfect and it is important to ask, now that many PDXs have been generated, how robustly can they represent the human disease? One limiting aspect of patient derived models is that after a maximum of a few passages the human stromal compartment has been completely replaced by mouse strome [15,51,64,65], making it difficult to study all cancer cell-stroma interactions due to the species-species differences. For example, the human Met receptor does not recognize the mouse met ligand so paracrine met signaling is not recapitulated. Also, mouse prolactin (PRL) antagonizes the human PRL receptor, thereby impairing the ability of PRL positive human tumors to grow in mice [66]. Additional concerns revolve around the high vascularization, which does not always reflect the vascularization state in humans, and lends to question if any anti-angiogeneic drugs can be evaluated in xenografts in a subcultaneous setting [67]. Another obvious feature is that the compromised immune system leads to issues in studying the response of immune oncology drugs. 

However, despite these obvious limitations of the models, some features do prove faithful to the human tumors. One of the initial observations that accelerated PDX models into the forefront over using cell line derived models was the notation of the recapitulation of the histology of the models, which prove to have sharp organizational structure and tissue architecture. PDX models generally retain the histopathological features seen in human tumors [51,68,69] contrast to cell line-derived xenograft models, which histologically cannot be differentiated. 

In addition to preserving the histopathological features of the original tumors, it is critical for any preclinical model to retain the molecular features to be clinically relevant [65,70]. Due to the long-term two-dimensional (2D) propagation on plastic of the generally available cell line-derived models, molecular divergence from the original tumors by selection under non-physiological conditions is evident [71,72]. To demonstrate that PDX models are indeed more faithful at the genetic and genomic levels, researchers have employed a number of approaches, including next-generation sequencing (NGS), gene expression profiling, and comparative genomic hybridization (CGH), to extensively profile these models. These studies generally show high levels of agreement between patient tumors populations and the PDX collections [12,15,73,74,75]. Indeed, an extensive study [1] where the molecular characteristics of over 300 PDX models were compared against The Cancer Genome Atlas (TCGA), found that the PDX populations much more faithfully represent the TCGA (i.e., patient populations) than the Cancer Cell Line encyclopedia (CCLE). The CCLE was found to have a preponderance of mutations in cell-cycle related genes. As well as the potential for selection pressures to be causative of these differences, in this comparison it should also be appreciated that PDX models and TCGA represent naive tumor populations, whereas the CCLE consists of many models from later stage disease that have previously been exposed to therapies, compromising the comparison It should also be noted that a new generation of cell line models are being created directly from patients and PDX models both in 2D and three-dimensional (3D) with multiple layers of complexity by many groups [26,76,77]; only time will tell how this new generation of cell lines or model systems such as organ-on-a-chip [78] represent the patient population as compared to PDXs, and if using PDX models in vivo will be surpassed in high-throughput studies with the relative ease and cost factors of in vitro experiments. 

Although the fidelity in molecular features in general has been well preserved in PDX models [35,79], the selection and clonal evolution are inevitable, as are stochastic events, both contributing to the PDX being established from a fraction of the fragment that is implanted. Indeed, even before these changes can occur, it must be assumed that the small piece that is implanted will have to be highly representative of the primary tumor if the PDX that results could represent the patient that it came from. What is potentially more important for drug discovery though, is that when a PDX is created that it represents a patient, somewhere, (not necessary the patient it originated from), and that panels of PDX that are created can represent the inter-heterogenetity of patient populations. However, it is also important for drug discovery and translatability that a PDX remains stable enough after establishment, at least in early passage numbers, for multiple use of the models and extrapolation back to original genetic data. 

An increasing number of publications are showing, that genetic changes occur from the original fragment implant to the established xenograft model. These changes occur during the initial establishment and during subsequent passaging of the model, leading to genetic differences between PDXs and the original tumors [1,80]. Eirew et al., reported that although PDXs are generally faithful molecularly, the initial engraftment, and subsequent propagation could impact on the genomic clonal architecture. By deep genome sequencing and single-cell sequencing, they showed that, in all cases examined, both primary and metastatic tumors undergo various levels of initial clonal selection and that the changes continued over time during subsequent propagation, the largest impact being changes from primary tumor to initial PDX establishment. Gao et al. [1], looked at RNA seq data across passage numbers and also showed that genetic changes occur. 

## 4. Use of PDXs as Preclinical Models 

It is certain that PDX models do change genetically as they are passaged, however, understanding the implications this has on functionality is the most critical question in determining how these models can be used in drug discovery. Gao et al. took a cautious approach, restricting their experiments over early passage number: Drug responses in experiments with PDX models over early passage numbers were not variable, suggesting that the implications on these genetic changes are small. Ben Davis et al. [81] took the RNA seq data from that publication and produced an algorithm to determine chromosome number alterations (CNAs). From there, they inferred these CNAs could impact drug response with a limited number of treatments tested (note, as only a few drug treatments are actually impacted, this argues the general robustness of the models). However, the data is difficult to interpret given CNA calls were made from PDX of earlier passage numbers than efficacy data, and if CNA dynamically changes during passage number, this cannot be correlated with the functional data of later passage numbers. 

Potentially, the most dramatic implication of the genetic differences seen from primary tumor to established PDX lie over concerns with the Avatar approach. This is the practice of using PDXs to pre-clinically guide treatment decisions for patients from whom the tumors were derived [16,82]. As patient surrogates, these avatar PDX models hope to represent a powerful tool for addressing individualized therapy for the patient from which the PDX model was created. For this paradigm to work, the patient’s PDX must establish in mice within the time window of the first therapy decision. Given that PDX models do not often grow in mice, and, if they do, can take months to establish, only certain types of cancer can benefit from this approach. The other major potential caveat, as already discussed, is that the biopsy of the tumor captured and grown in the mouse may or may not represent the bulk of the tumor growing in the patient. Divergence from the original tumor could occur if the disease is very heterogeneous, and the fragment implanted does not faithfully representing the bulk of the tumor, or due to differential clones growing out, which also fail to capture the original state. However, the documented success of avatars, such as in ovarian and breast cancer [44,83] may suggest that the approach is possible and that issues may only arise in certain situations particularly when the disease state is very heterogeneous i.e., an issue for certain cancers at particular stages of diagnosis. 

Despite reports of avatar success, it is certainly true that not all PDXs represent the patient’s tumor they were derived from. Warm autopsy biopsies from different metastatic sites from the one patient can help us understands the issue of heterogeneity. Indeed, the analysis of melanoma PDXs from vemurafenib therapy-refractory metastases in a patient revealed that multiple resistance mechanisms were present within one metastasis and between metastases. It is also of note that this heterogeneity was not completely captured within the PDXs created from the different sites [84]. Further studies like these may enable a greater prediction to which avatars could be beneficial/predictive for patients, i.e., which types of tumor and at which stage. 

## 5. Using PDX in the Population Approach 

The most prolific use of PDX models used now though, by-passes the concern of the PDX needing to match the genotype from the patient the PDX is derived *per se*; the PDX model just needs to represent a patient. The preference of many in the last few years has moved from using one or a small selection of PDX models for drug discovery and translatability to using panels of PDX to capture the inter-heterogeneity of disease [10,12,15,85,86]. Here, studies are designed to predominantly attempt to mimic phase II clinical trials where typically dozens of people are enrolled for each trial. In this paradigm, few mice (usually just one) are used, bearing one type of PDX and given one treatment [1,10,12,86,87]. When looking at responses in this paradigm, it is not the response of an individual mouse/tumor that is important, but the population response, just like in a human clinical trial. Data is evaluated, close to how one would in a human clinical trial, for example, measuring “best response” and “time to progression”. It should be noted however that to completely capture the full inter tumor heterogeneity of a particular tumor type with various disease stages and prior treatments represented, large panels are necessary. This is not feasible for tumor types that poorly establish. 

This type of large-scale screening using the “mouse clinical trial” or MCT approach was first described by Migliardi et al. in 2012 [86]. Migliardi et al. chose to evaluate the effect of four different treatment arms (ERK, MEK, and PI3K inhibitors) across 40 different colon cancer PDXs each with an *n* = 4. Another study [87], Bertotti et al., took a similar screening approach, in this case using over 100 different colorectal cancer (CRC) models (*n* = 5 or 6) to profile the efficacy of epidermal growth factor receptor (EGFR) inhibitor, cetuximab, and found concordance in the response to this drug to EGFR-amplified models and CRC patients in the clinic. In the studies by note by Townsend et al. and Gao et al. [1,10] just one tumor representing “a patient” was used to allow for even greater efficiency (the so-called 1 × 1 × 1 approach). In both cases, investigators determined that using the one animal per cohort study design has outstanding reproducibility for data collected. A very comprehensive study in a panel of pediatric tumors [88] substantially analyzed the individual tumor response by taking a randomly chosen mouse and compared the response to the group median. Allowing for the misprediction of +/− one response category (stable disease, partial response, complete response), the overall mean correct single mouse prediction rate was 95.28% and predicted overall object response rates for group data in 66 of 67 drug studies. The ability to use the 1 × 1 × 1 approach of course enables many more types of PDX and treatment groups to be assessed operationally, and the inter-heterogeneity of patients to be captured experimentally. This has been most comprehensively demonstrated in a study where 62 treatments were assessed across six indications comprised of 29–45 models per indication [1]. The MCT approach has also proven to be elucidate potentially promising drugs in a “phase II” like study using B-ALL (B-cell acute lymphoblastic leukemia) PDXs 10 pediatric studies [12,88], and a breast PDX panel [15] among others. Consequently, this approach has now been adapted by many pharmaceutical companies [89], many of whom use CROs with extensive PDX collections to perform such studies. 

The power of looking at population response, i.e., assessing inter-tumor heterogeneity, enables assessment of the effect a drug may have across the diseases state, as opposed to the traditional methods of assessing drugs in just a few models, which, depending on the models chosen, may under or over predict a response across many. Hopefully, this methodology will increase success rates in the clinic; currently less than 10% of oncology drugs entering clinical trials ultimately get approved [60,70]. The power of being able to identify responsive subpopulations i.e., assessing inter-tumor heterogeneity, enables assessment of the effect a drug may have across the disease state, as opposed to the traditional methods of assessing drugs in just a few models, which depending on the models chosen, may under or over predict a response across many. Hopefully this methodology will increase success rates in the clinic; currently less than 10% of oncology drugs entering clinical trials ultimately get [92,93]. Being able to identify responsive subpopulations can focus clinical trials to where they could be most beneficial and the mouse clinical trials have led to the discovery of predictive biomarkers, both signatures and select markers of known mechanism of action for a wide variety of small molecule inhibitors [93,94]. For antibody drug conjugates (ADCs), panels of tumors with different expression levels can help to determine the intensity and homogeneous expression of an antigen should be to see response [94]. The variety of the panel of PDX models can also help choose linkers and payloads for ADCs that maybe to most universally effective across the disease [95]. 

Another interesting use of the un-bias mouse clinical trial approach is the ability to discover novel biology, and therefore position drugs in patient populations not previously considered. A recently published example of this involved the efficacy of the cdk4/6 inhibitors, known to be involved in cell cycle arrest in retinoblastoma protein (RB1) intact cells. The mouse clinical trials revealed that in select models, dramatic apoptosis occurs—and these and other studies revealed that cyclin D3-cdk6 have RB1 agnostic substrates involved in metabolism pathways, leading to a novel mechanism of action of this compound and a potential new sub-set of patients to treat [96]. 

Retrospective translatability of mouse clinical trials suggests they are able to enhance the predictable nature of clinical trial, not just with efficacy but also in the ability to identify clinically relevant mechanisms of resistance [1].

The wealth of data that can be obtained by the panel approach has also meant it has been used as a source of data to complement clinical trial data when testing out hypotheses, for example, in regards to how mechanistically combinations effect efficacy [97], leading again to concordance between clinical trials and MCT and adding to the confidence of this pre-clinical approach. 

Most anticancer drug candidates entering clinical development are tested in late-stage cancer patients who have previously failed several lines of chemo- and/or targeted therapies. It is essential that the appropriate PDXs be selected to reflect the desired disease stage for a certain therapy [98,99]. The majority of the first panels of PDX models created were from tumor specimens from treatment naïve patients due to the ease of access of biopsies from first diagnosed patients and the resection of their disease. However, due to the recognized importance of samples from late stage patients, and the need to test potential drugs in the PDXs most likely to model the clinical trials they will be tested in, had led to a concerted effort to produce panels of treatment refractory tumor PDX models and this resource has been growing It should be noted that whether or not the models that are established from clinically refractory tumors, remain refractory to the same treatment in the mouse has not been well documented. 

## 6. Summary 

In summary, PDX models continue to be a preferred model for drug discovery and translation, despite some of their limitations. The range and breath of the available PDX models that are now available are powerful, as they capture much of the inter-patient heterogeneity seen across patients. The data produced from the PDX panels enables patient stratification strategies, identifies novel predictive biomarkers and can uncover new biology not hypothesized previously. The potential ability to combine a human immune system with the PDX models to enable the assessment of many immunomodulatory agents (as single agents and in combination with targeted therapies) across a diverse range of models remains attractive, and dependent on increasingly sophisticated mouse strains. The understanding of how novel 2D and 3D ex vivo assays perform relative to in vivo PDX modelling is also in its infancy with the possibility of creating more efficient drug screening. 
